# Access to Chemical Data Used in Regulatory Decision Making

**DOI:** 10.1289/ehp.1206438

**Published:** 2013-04-01

**Authors:** Lynn R. Goldman, Ellen Silbergeld

**Affiliations:** 1 George Washington School of Public Health and Health Services, Washington, DC, E-mail: goldmanl@gwu.edu; 2 Department of Environmental Health Sciences, Bloomberg School of Public Health, Johns Hopkins University, Baltimore, Maryland

It is clear from our commentary (Goldman and Silbergeld 2013), that we disagree with Lutter et al. (2013) about whether the public disclosure of all raw data used by the U.S. Environmental Protection Agency (EPA) for making regulatory decisions for chemicals is necessary to ensure the scientific basis for such decisions, and about the extent to which pre-emptive dis-closure (prior to any request) is practical. However, the most important disagreement between us is the basis asserted by Lutter et al. in their commentary for this change in policy. Lutter et al. argued that it is necessary for the U.S. EPA—and anyone else who desires to do so—to reanalyze all data used in their assessments in order to “replicate” the findings and conclusions of the original investigators.

Lutter et al. (2013) repeatedly used the terms “replicability” and “replication” as synony-mous with an “independent analysis” of raw data from an existing study. Replication in science is quite different; it involves performance of an independent study with the same hypothesis and then testing the extent to which this independent study reaches the same conclusions. Recalculation of study statistics or other reanalysis of an existing study data set is not a replication. Designing and conducting a replication study does not require access to raw data from the original study; this would abrogate the concept of independence. Moreover, an independent study will by defini-tion utilize different sets of animal models or human populations, and as a consequence may employ different statistical techniques.

Their second argument is that disclosure of raw data will assist in identifying sources of scientific bias. We consider this unlikely because the most important sources of bias are usually related to problems in study design or limitations of the data collected. This is not identifiable through data recalculation; however, this type of bias can usually be identified in the text of the original study publication.

Lutter et al. (2013) noted (correctly) that applicants to the U.S. EPA for pesticide regis-trations must provide raw data from regulatory testing as part of the package submitted to the U.S. EPA. This is a very special case, in that these studies are neither peer reviewed nor accessible to the public because of the protection sought by industry and extended by law for confidential business information (CBI). The assumption of bias related to these studies is not unreasonable, given that they are conducted by or on behalf of commercial entities seeking to obtain pesticide registration. These studies are rarely published in the scien-tific literature or in any way subject to independent peer review other than review by the U.S. EPA. Many scientists and public policy practitioners consider the CBI cloak as a major impedi-ment to transparency and confidence. Industry could demonstrate their commitment to transparency by declining this protection, thereby increasing the confidence of all.

Finally, Lutter et al. (2013) attempted to support their proposal by claiming that journals [*Nature* and the *Proceedings of the National Academy of Sciences of the United States* (*PNAS*)] and an expert body (the Bipartisan Policy Center) agree with them. However, these bodies have neither supported the concept of requiring that all raw data be reported to the the U.S. EPA nor that the U.S. EPA carry out its own independent recalculation. Rather, *Nature* and *PNAS* require authors to agree to make data sets (as well as materials and protocols) available to editors, and to others, upon request (Nature Publishing Group 2012; PNAS 2012). One of us (L.R.G.) was a member of the Science for Policy Project; its final report (Bipartisan Policy Center 2009) also recommended this practice. Many journals require data, such as DNA and protein sequences, macro-molecular structures, micro-array data, and crystallo-graphic data, to be made available on publicly accessible databases, but most of these are not “raw data” in the sense that Lutter et al. proposed. *Nature* also recommends that authors submit clinical trials data to external clinical trials databases (Nature Publishing Group 2012).

In summary, we disagree with the argu-ment that raw data from every study used by the U.S. EPA to support a regu-la-tory assess-ment should be made available to the agency and to the public. This proposal does not serve the purpose of “replication” or identi-fica-tion of bias, as asserted by Lutter et al. (2013). In practice, it may generate obstacles to good science and discourage researchers from studying issues of importance in environ-mental health. This proposal would also limit the U.S. EPA from using the results of research published in the peer-reviewed scientific litera-ture by placing studies off-limits if the authors did not submit raw data sets to the the U.S. EPA.

Finally, there is no obvious need for these changes. When the U.S. EPA has determined a need to reanalyze data, the current regulatory practice has not impeded such activities. Past history indicates that difficult cases are rare and do not warrant an intrusive and burden-some new requirement for the automatic submission of data from all studies.
